# Supplementary material for: Transcriptomic analysis and physiological characteristics of exogenous naphthylacetic acid application to regulate the healing process of oriental melon grafted onto squash
Source: PeerJ. 2022 Sep 15;10:e13980. doi: 10.7717/peerj.13980 (PMC9482769; doi:10.7717/peerj.13980)
Supplement: Supplemental Information 1 [file peerj-10-13980-s001.docx]

Table S1 The list of specific primers used in fluorescence quantitative PCR detection

| Gene ID | （Primer sequences） |
| --- | --- |
| *CmoCh08G003030* | (F) CAAGACTGCCCTTGTAACG  (R) GTCCACTTTACGCAGATATGG |
| *CmoCh07G009530* | (F) GATGTGATGGTCGAGTTGG  (R) GAGCTCCACCTTGATTGG |
| *CmoCh20G005830* | (F) GACAAGAACACCGAGAAGG  (R) CGTGCCCTTTGACTTCC |
| *CmoCh08G005650* | (F) CGGCGATGTTTACTCATACC  (R) CATAATAGCACCGCGATACG |
| *MELO3C014091.2* | (F) AATTCCGGATGATGGAAAGG  (R) CAGCTTTGGTTGCTAGGG |
| *MELO3C004382.2* | (F)CGCAACAACCTAACGATT  (R)GACGACGACACTCCTAAG |
| *MELO3C015359.2* | (F) GCAAACAAAGTGCAGATGG  (R) TTGAGGCATTGGCATCC |
| *MELO3C034560.2* | (F) GCTGTTCATCCTCTTGATCC  (R) GTCCTCCAAAGTTGCTTCC |
| *MELO3C010317.2* | (F) TGAAGATGGAGGGAGTGG  (R) TTCGTTCTCAGCAAGTAACC |
| *MELO3C026019.2* | (F) ACCGACTCGCTAACTACC  (R) GACTGAAGGTTGCATCGG |
